# Cardiovascular Diseases in Indigenous Populations: An Indicator Of Inequality

**DOI:** 10.5935/abc.20180045

**Published:** 2018-03

**Authors:** Airton Tetelbom Stein

**Affiliations:** Departamento de Saúde Coletiva da Universidade Federal de Ciências de Saúde de Porto Alegre (UFCSPA), Porto Alegre, RS - Brazil; Grupo Hospitalar Conceição, Porto Alegre, RS - Brazil

**Keywords:** Cardiovascular Diseases / mortality, Indigenous Population, Urbanization / trends, Health Care (Public Health), Social Change, Risk Factors

The number of articles on cardiovascular diseases in indigenous populations is not sufficient as a basis for the development of health policies. Although the most common conditions reported among indigenous people have been infectious and contagious diseases such as malaria, tuberculosis, respiratory infections, hepatitis, sexually transmitted diseases, among others, the prevalence of noncommunicable diseases (NCDs) have increased in this population as a result of urbanization process and their lifestyle. In addition, approaches of these conditions in indigenous people face logistic issues, as they require continuous medical care and development of health promotion programs in difficult areas.

The original, well-conducted study by Armstrong and colleagues contributes to fill this knowledge gap, as it highlights the impact of public investments that not only promote the development of the country, but also reveals the vulnerability and adverse effects of the changes in the lifestyle of indigenous people. Despite its methodological limitations, the study shows an association between mortality for cardiovascular diseases and rapid urbanization in this population.^1^

This phenomenon has not been described only in Brazil. A study conducted on indigenous people in the southeastern Asia described the influence of the urbanization process on epidemiological transition and increase in NCDs.^2^

Life expectancy and disease rates are variable and dependent on demographic and geographical characteristics of where people live.^3^ The health system has a key role to reduce inequality; in this context, it is essential to implement intersectoral interventions at community level, particularly due to limited resources and need for effective interventions.

Community and family physician, together with other healthcare professionals, should foster indigenous traditional culture and integration of indigenous people to society as urbanization of their communities occurs. Primary health care should seek to identify and develop strategies in relation to social determinants, relevant to both communicable and NCDs.^4^

The context encountered by healthcare professionals in indigenous communities is a high prevalence of risk factors for NCDs, including overweight, smoking, alcohol consumption and unhealthy diet. This situation, which results from epidemiologic transition, is even more critical in these people, because of the occurrence of infectious diseases.

The classical study by Geoffrey Rose^5^ is still current and points out the necessity to identify the cause of the causes, especially in case of a rapid urbanization without a careful planning, which leads to a worse quality of life associated with the stress of new challenges and exposure to risk factors for cardiovascular diseases.

Therefore, the present study shows the vulnerability of the indigenous people, whose health conditions require planning by health system managers. This should consider the difficulty of this population to integrate into society and have access to health services, which, in turn, should be prepared to meet their needs.

Health care services for indigenous communities should be aware of the high rates of unhealthy behavior and adverse social conditions related to an unhealthy environment - aspects that leads to inequality - as occurs in San Francisco Valley in the northeast of Brazil.

Increasing number of interventions on NCDs aimed at achieving Sustainable Development Goals have been described, and publication of their results in academic scientific literature should be highly encouraged to promote best practice.^6^

The role of journals like *Arquivos Brasileiros de Cardiologia* is to inform the frequency of cardiovascular diseases, to report etiologic, diagnostic, and prognostic approaches to these conditions, as well as the most effective interventions that should be encouraged by health managers. This is particularly relevant in the indigenous population, which is likely to experience an increase in the incidence of cardiovascular diseases as a reflection of urbanization. The actions developed by the government, especially in indigenous areas, should encompass intersectoral actions to reduce health problems and inequalities in this population.
